# Gene Overexpression Resources in Cereals for Functional Genomics and Discovery of Useful Genes

**DOI:** 10.3389/fpls.2016.01359

**Published:** 2016-09-21

**Authors:** Kiyomi Abe, Hiroaki Ichikawa

**Affiliations:** Institute of Agrobiological Sciences, National Agriculture and Food Research OrganizationTsukuba, Japan

**Keywords:** cereals, rice, transgenic plants, resources, overexpression, FOX hunting, chimeric repressors, activation tagging

## Abstract

Identification and elucidation of functions of plant genes is valuable for both basic and applied research. In addition to natural variation in model plants, numerous loss-of-function resources have been produced by mutagenesis with chemicals, irradiation, or insertions of transposable elements or T-DNA. However, we may be unable to observe loss-of-function phenotypes for genes with functionally redundant homologs and for those essential for growth and development. To offset such disadvantages, gain-of-function transgenic resources have been exploited. Activation-tagged lines have been generated using obligatory overexpression of endogenous genes by random insertion of an enhancer. Recent progress in DNA sequencing technology and bioinformatics has enabled the preparation of genomewide collections of full-length cDNAs (fl-cDNAs) in some model species. Using the fl-cDNA clones, a novel gain-of-function strategy, Fl-cDNA OvereXpressor gene (FOX)-hunting system, has been developed. A mutant phenotype in a FOX line can be directly attributed to the overexpressed fl-cDNA. Investigating a large population of FOX lines could reveal important genes conferring favorable phenotypes for crop breeding. Alternatively, a unique loss-of-function approach Chimeric REpressor gene Silencing Technology (CRES-T) has been developed. In CRES-T, overexpression of a chimeric repressor, composed of the coding sequence of a transcription factor (TF) and short peptide designated as the repression domain, could interfere with the action of endogenous TF in plants. Although plant TFs usually consist of gene families, CRES-T is effective, in principle, even for the TFs with functional redundancy. In this review, we focus on the current status of the gene-overexpression strategies and resources for identifying and elucidating novel functions of cereal genes. We discuss the potential of these research tools for identifying useful genes and phenotypes for application in crop breeding.

## Introduction

Modern plant breeding has developed several key technologies in crop breeding that have contributed to increasing and sustaining food production. Among the key technologies, the most noteworthy is the development of high-yielding and semi-dwarf varieties in rice and wheat between the 1940s and late 1960s. This technology led to increased agricultural production and is known as the Green Revolution (Khush, 1999; Basu et al., 2010). Because the improved varieties were resistant to lodging and were grown with appropriate chemical fertilization, they showed increased productivity per unit area. A second important key technology is the development of F1 hybrid varieties. Particularly, in the United States, production of hybrid maize spread widely and rapidly, and hybrid varieties became substitutes for open-pollinated varieties in 1940s. The productivity per unit area of the hybrid maize has dramatically increased: five times greater than that of pre-hybrid varieties (Crow, 1998).

Plant breeding by genetic engineering was developed in the 1980s and has become the third key technology. For molecular breeding, accumulation of molecular biological information and resources on crops is of great value. DNA sequencing technology and bioinformatics continue to evolve and whole-genome sequencing has been accomplished in maize and wheat, following that of rice (Schnable et al., 2009; Brenchley et al., 2012; International Wheat Genome Sequencing Consortium (IWGSC), 2014; Michael and VanBuren, 2015). Exploiting this information for molecular breeding will depend on the identification and characterization of functions of cereal genes. For this purpose, various collections of plant resources and their databases (DBs) have recently been developed for rice and other cereals (Mochida and Shinozaki, 2010; Tsuchida-Mayama et al., 2010; Kersey et al., 2016; Tello-Ruiz et al., 2016).

Plant genetic resources artificially induced by various mutagens can be classified into two groups based on the types of mutation: loss- and gain-of-function resources. Production of loss-of-function resources for cereals became active at the end of the 20th century after the effectiveness of ionizing radiation, alkylating chemicals and various genetic elements as potent mutagens was established (Kurata et al., 2005; Krishnan et al., 2009; Morita et al., 2009; Satoh et al., 2010). Gain-of-function resources for cereals have become popular in the 21st century in coordination with the development of transformation technology (Hiei et al., 2014) and genomics, including genome sequences, and the information and clones for full-length cDNAs (fl-cDNAs) (Tsuchida-Mayama et al., 2010; Abdeeva et al., 2012). In this review, the current status of these resources developed for identifying and deciphering novel functions present in numerous cereal genes will be overviewed with emphasis on overexpression resources. Also, the potential of these research tools for identifying useful genes and phenotypes for application in crop breeding will be evaluated and discussed.

## Loss-of-function Resources By Direct Modification of Genes

Loss-of-function resources can be classified into two groups. The first group of resources is induced by irradiation with physical mutagens including X-rays, γ-rays, fast neutrons (FNs), and heavy ions (ion beams), and treatment with chemical mutagens such as ethyl methanesulfonate (EMS) and *N*-methyl-*N*-nitrosourea (MNU). These mutagens are applicable to any species regardless of the availability of efficient transformation systems. Therefore, using these mutagens, numerous mutant populations of cereals have been produced and characterized with the aim of isolating genes responsible for respective mutations (Kurata et al., 2005; Wu et al., 2005; Morita et al., 2009; Yamaguchi et al., 2009; Jiang and Ramachandran, 2010; Satoh et al., 2010; Kurowska et al., 2012). Mutants with easily observable phenotypes have been screened from the mutagenized populations; the causative genes have then been identified by map-based cloning strategies. Causative genes have been genetically confirmed by complementation tests. Using this workflow, various cereal genes have been identified and functionally characterized using rice mutants (Huang et al., 2009; Gao et al., 2010; Jiang and Ramachandran, 2010; Yang et al., 2011).

New reverse genetic methods have recently been developed to identify and isolate mutants for individual target genes from chemically or physically mutagenized populations (Jiang and Ramachandran, 2010; Wang et al., 2012). Chemical reagents, such as EMS and MNU, efficiently induce chemical modification of nucleotides, resulting in various point mutations. Targeting Induced Local Lesions In Genomes (TILLING), a reverse genetic method for high-throughput identification of single-nucleotide polymorphisms (SNPs) in a target gene, is applicable to the mutagenized populations with point mutations and short insertions and deletions (INDELs) (Till et al., 2003; Wang et al., 2012). Suzuki et al. (2008) modified the TILLING system using unlabeled primers and fast capillary gel electrophoresis and efficiently detected SNPs in a mutant library of rice using individual zygotes treated with MNU to produce a high mutation rate: one mutation per 135 kbp of genome sequence (1/135). A frequency of 7.4 SNPs per 1 kbp of genome sequence was estimated for 1,000 M2 mutant lines of rice. A diploid einkorn wheat (*Triticum monococcum*), the first domesticated crop, is an attractive genetic model for studying the functions of genes conferring wheat-specific traits. Rawat et al. (2012) prepared 1,532 M2 families of the diploid wheat induced by EMS for TILLING analysis. The estimated mutation density was 1/92 kbp. Compared with this rate, the rates for hexaploid bread wheat (*T. aestivum*) were much higher [1 per 24–47 kbp; Chen et al., 2012; Rawat et al., 2012] because of the higher tolerance of polyploids to mutations. FNs with relatively high linear energy transfer (LET) can induce kilobase-scale deletions in plant genomes ranging from a single base to 60 kbp (Rogers et al., 2009; Shu et al., 2012). Compared with mutations produced by chemical mutagenesis, such deletions tend to produce null mutations with few background mutations. Reverse-genetic procedures, consisting of FN mutagenesis of a population followed by high-throughput PCR screening of the population, detected deletions in *Arabidopsis* and rice (Li et al., 2001) and *Medicago* (Rogers et al., 2009).

The procedures are convenient and powerful if the following conditions are met: large populations to be prepared because of low FN mutation frequencies and deletions must be of appropriate size to be flanked by designed PCR primers that generate mutant amplicons separable from those of the wild-type (Till et al., 2007). Recent findings of Morita et al. (2009) and Belfield et al. (2012) indicated that γ-rays and FNs induce base substitutions and deletions of a few bases more frequently than larger-scale deletions in rice and *Arabidopsis*, respectively. Bruce et al. (2009) applied oligonucleotide microarray-based comparative genomic hybridization (array-CGH) to identify both small and large deletions in rice lines irradiated with FNs and γ-rays. Belfield et al. (2014) identified, using array-CGH, small deletions (4–104 bases) in FN-irradiated mutants of *Arabidopsis*. Nishizawa-Yokoi et al. (2016) screened, with TILLING, 1,968 lines of gamma-irradiated rice for a defect in the *DNA Ligase4* (*OsLig4*) locus and obtained an *Oslig4* mutant bearing a 35-base deletion in exon 14. To retrieve mutants among soybean plants heavily mutagenized with EMS, Tsuda et al. (2015) designed and compared two novel screening strategies: high resolution melting (HRM) assay and indexed amplicon sequencing [DNA sequencing by next-generation sequencing (NGS) technologies of PCR fragments for target gene regions amplified from pooled samples], and reported that the latter detected mutated targets more efficiently.

The second group of resources is derived by insertion of endogenous or exogenous DNA insertion elements to generate loss-of-function mutations. In rice, large loss-of-function mutant populations have been generated by activation and random genome insertion of the *Tos17* retrotransposon (Hirochika, 2001; Miyao et al., 2003, 2007), *Ds* and *dSpm* transposable elements (Upadhyaya et al., 2002; Kolesnik et al., 2004; Kumar et al., 2005; van Enckevort et al., 2005; Park et al., 2007), or T-DNAs (An et al., 2003; Chen et al., 2003; Ryu et al., 2004; Sallaud et al., 2004). These loss-of-function mutant resources are convenient for identification and functional analyses of genes disrupted and tagged by insertion of the DNA elements and can be widely used for both forward and reverse genetic approaches.

Ogawa et al. (2011) isolated a dwarf and short-root mutant (*rice salt sensitive 1, rss1*) under high salt stress among *Tos17*-mutagenized rice plants (M2 generation from ∼2,500 lines). They obtained two more alleles of *rss1* mutation by PCR-based screening of DNA pools of genomic DNA mixtures from *Tos17*-insertion lines. Molecular analysis indicated that *RSS1* encodes a novel protein that acts as a key factor in the maintenance of meristematic activity by ensuring cell division under saline conditions (Ogawa et al., 2011).

Zou et al. (2011) screened more than 100,000 T-DNA insertion lines of rice derived from several populations and isolated 312 mutant lines with rolled leaves. Among them, an *outcurved leaf1* mutant showing abaxial (outside) leaf rolling was identified. This phenotype was caused by knockout of *Rice outermost cell-specific gene5* (*Roc5*). Overexpression of *Roc5* induced an opposite phenotype, adaxially rolled leaves, to the *Roc5*-knockout rice. *Roc5* encodes a homeodomain leucine-zipper class IV TF, an ortholog of *Arabidopsis GLABRA2*. Further analyses indicated that Roc5 functions as a negative regulator controlling bulliform cell number and size in rice leaves (Zou et al., 2011). Ning et al. (2011) found a transgenic rice plant bearing a T-DNA insertion in the fourth exon of *Increased Leaf Angle1* (*ILA1*) gene among a large T-DNA-insertion population of more than 130,000 lines developed for enhancer tagging (Rice Mutant Database^1^). The *ila1* mutant displayed smaller vascular bundles and reduced cellulose content in leaf lamina joints, and the phenotypes were unrelated to response to brassinosteroids (BRs). Molecular analyses revealed that *ILA1* encodes a Raf-like MAPKKK and is mainly expressed in vascular bundles of lamina joints. Ning et al. (2011) identified a novel mechanism of leaf angle regulation by ILA1, a key factor conferring mechanical strength to lamina joints. Ramamoorthy et al. (2011) screened visible mutant lines among approximately 20,000 lines of rice carrying *Ds* insertions and found a mutant with semi-dwarfism caused by defects in cell elongation. The mutant line included a single-copy *Ds* insertion in *OsCYP96B4*, a member of the cytochrome P450 multigene family.

## Gain- and Loss-of-function Resources By Overexpression

**FIGURE 1:**
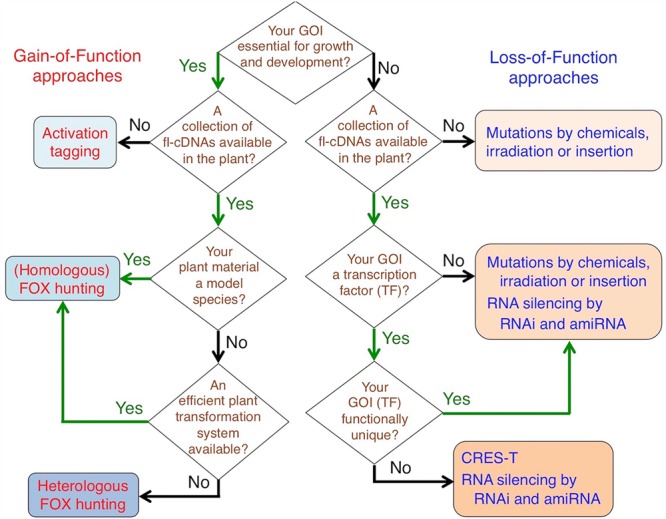
**Choosing appropriate resources to isolate your gene(s) of interest by forward and/or reverse genetic approaches.** GOI, Gene(s) of interest.

As described in the previous section, many mutants have been isolated from loss-of-function resources and their causative genes have been identified and characterized. However, it would be difficult to observe loss-of-function phenotypes for gene families with functional redundancy, such as those for various TFs and genes essential for growth and development. To address this disadvantage, various overexpression systems for transgenes have been developed: gain-of-function resources and tools based on activation tagging and Full Length-cDNA OvereXpressor gene (FOX) hunting, and loss-of-function resources and tools on gene-silencing technologies with RNA interference (RNAi) and artificial microRNAs (amiRNAs), and on a dominant-negative strategy, Chimeric REpressor gene Silencing Technology (CRES-T) (Walden et al., 1994; Horiguchi, 2004; Eamens et al., 2008; Tsuchida-Mayama et al., 2010; Kondou et al., 2011; Mitsuda et al., 2011a; Abdeeva et al., 2012; Sun, 2012; Zheng and Qu, 2015). Researchers may select these resources depending on their research purposes (**Figure 1**). In the following paragraphs, transgenic resources based on both gain- and loss-of-function approaches (**Table 1**) and also some examples of characterization and application of gene functions in cereals (**Table 2**) are introduced.

**Table 1:** Loss- and gain-of-function transgenic resources in cereals.

Resource no.	Mutagen/Transgene^∗^	Classification^∗∗^(Loss or gain of function)	Host plant(cultivar, ecotype)	Population size	Reference
1	T-DNA (GT)	Loss	Rice (Dongjin, Hwayoung)	20,810	Jeong et al., 2002; An et al., 2003
2	*Ac/Ds* in T-DNA (GT, ET)	Loss	Rice (Nipponbare)	∼16,000 as mutagenized loci^∗∗∗^	Upadhyaya et al., 2002; Eamens et al., 2004
3	*Ac/Ds* in T-DNA (GT)	Loss	Rice (Dongjin, MGRI079)	95,900 regenerated plants from *Ac/Ds* cross combinations	Kim et al., 2004; Park et al., 2007
4	*Ac/Ds* in T-DNA (GT)	Loss	Rice (Nipponbare)	∼20,000 F2 families from 25 *Ac/Ds* cross combinations	Kolesnik et al., 2004; Jiang et al., 2007
5	*Spm/dSpm* in T-DNA (GT)	Loss	Rice (Nipponbare)	9,036^∗∗∗∗^	Kumar et al., 2005
6	*Ac/Ds* in T-DNA (GT)	Loss	Barley (Golden Promise)	4,954 F2 populations from 8 *Ac* x *Ds* cross combinations	Brown et al., 2015
7	T-DNA (ET)	Loss	Rice (Nipponbare)	29,482	Sallaud et al., 2004
8	T-DNA (ET)	Loss	Rice (Nipponbare)	∼100,000	Yang et al., 2004
9	T-DNA (ET)	Loss	Rice (Zhonghua 11, Zhonghua 15, Nipponbare)	128,560	Zhang et al., 2006
10	*Ac/Ds* in T-DNA (ET)	Loss	Rice (Nipponbare, Pusa Basmati, Bengal)	∼25,000 as mutagenized loci^∗∗∗^	van Enckevort et al., 2005
11	T-DNA (PT, AT)	Loss and Gain	Rice (Tainung 67)	55,000	Hsing et al., 2007
12	T-DNA (AT)	Gain	Rice (Dongjin, Hwayoung)	47,932	[Bibr B49][Bibr B50]
13	T-DNA (AT)	Gain	Rice (Nipponbare)	∼13,000	Mori et al., 2007
14	T-DNA (AT)	Gain	Rice (Nipponbare)	∼50,000	Wan et al., 2009
15	T-DNA (AT)	Gain	Rice (Zhonghua 11, Chaoyou 1, Taichung 65, Nipponbare)	>200,000	Lu et al., 2014
16	*Ac/Ds* in T-DNA (AT)	Gain	Barley (Golden Promise)	60,000 F2 plants from 35 *Ac/Ds* cross combinations	[Bibr B6][Bibr B5]
17	T-DNA (rice RIKEN-FOX)	Gain	Rice (Nipponbare)	11,582	Nakamura et al., 2007
18	T-DNA (rice FAIS-FOX)	Gain	Rice (Nipponbare)	2,586	Hakata et al., 2010
19	T-DNA (rice *CDPK*-FOX)	Gain	Rice (Nipponbare)	250	Asano et al., 2011
20	T-DNA (rice RIKEN-FOX)	Gain	*Arabidopsis* (Columbia)	23,715	Kondou et al., 2009
21	T-DNA (wheat TF-FOX)	Gain	Rice (Nipponbare)	>15,000	Wu et al., 2015

*^∗^Abbreviations: GT, gene trap; ET, enhancer trap; PT, promoter trap; AT, activation tagging; TF, transcription factor. ^∗∗^Classified according to the genotype at a specific locus with T-DNA insertion. ^∗∗∗^Adapted from Zhu et al. (2012). ^∗∗∗∗^Adapted from Krishnan et al. (2009).*

**Table 2:** Examples on the identification and characterization of genes by using transgenic resources in cereals.

Classification of resource (Loss- or gain-of-function)	Mutagenized or generated by	Host plant	Resource no. in Table 1	Genetic approach(Forward or Reverse)	Identified gene	(Plausible) function(s) of the gene product	Reference
Loss-of-function	T-DNA insertion	Rice	1, 7	Reverse	*STR1*	Stunted arbuscule (STR) 1 protein belongs to G subfamily of the half-size ABC transporters; mycorrhizal arbuscule formation	Gutjahr et al., 2012
Loss	T-DNA insertion	Rice	1	Forward	*MPK6*	Mitogen-activated protein kinase; cell differentiation during early embryo development	Yi et al., 2016a
Loss	T-DNA insertion	Rice	1	Forward	*DTC1*	DTC1 contains a development and cell death (DCD) domain and KELCH repeats; key regulator for programmed cell death of tapetum by inhibiting ROS-scavenging activity	Yi et al., 2016b
Loss	T-DNA insertion with Ac/Ds	Rice	2	Forward	*AID1*	A telomere-binding protein with a MYB-like domain at the C-terminus (AID family); anther development with pleiotropic effects on tillering and flowering time	Zhu et al., 2004
Loss	T-DNA insertion with Ac/Ds	Rice	3	Forward	*CSLD1*	A cellulose synthase-like D1 protein; root hair elongation	Kim et al., 2007
Loss	T-DNA insertion with Ac/Ds	Rice	4	Forward	*CYP96B4*	Cytochrome P450 (CYP96 subfamily) protein; regulating cell elongation and pollen germination through lipid metabolism	Ramamoorthy et al., 2011
Loss	T-DNA insertion with Spm/dSpm	Rice	5	Reverse	*PT13*	PHOSPHATE TRANSPORTER1 (PHT1) family protein conserved in monocots; regulation of arbuscular mycorrhizal (AM) symbiosis	Yang et al., 2012
Loss	T-DNA insertion with Spm/dSpm	Rice	5	Reverse	*STR2*	Stunted arbuscule (STR) 2 protein belongs to G subfamily of the half-size ABC transporters; mycorrhizal arbuscule formation	Gutjahr et al., 2012
Loss	T-DNA insertion	Rice	7	Reverse	*SERF1*	A salt-responsive TF of AP2/ERF family; Positive regulator of salt stress tolerance	Schmidt et al., 2013
Loss	T-DNA insertion	Rice	8, 14	Forward	*OUL1/Roc5*	HD-Zip IV TF; negative regulator controlling bulliform cell number and size in leaves	Zou et al., 2011
Loss	T-DNA insertion	Rice	9	Forward	*ILA1*	Raf-like MAPKKK; key factor conferring mechanical strength to lamina joints	Ning et al., 2011
Gain-of-function	Activation tagging	Rice	12	Reverse	*COL4*	CONSTANS-like (COL) family protein; flowering repressor	Lee et al., 2010
Gain	Activation tagging	Rice	12	Reverse	*YSL16*	A member of the Yellow Stripe1-Like (YSL) family of transporter protein; iron transport	Lee et al., 2012
Gain	Activation tagging	Rice	13	Forward	*SPL18/AT1*	Acyltransferase; lesion mimicry and disease resistance	Mori et al., 2007
Gain	Activation tagging	Rice	13	Forward	*SG1*	A protein conserved in angiosperms; organ elongation and brassinosteroid response	Nakagawa et al., 2012
Gain	Activation tagging	Rice	15	Forward	*GLR1, GLR2*	Glutamate receptor-like proteins; tolerance to drought stress	Lu et al., 2014
Gain	Activation taging with Ac/Ds	Barley	16	Forward	*Uros*	Uroporphyrinogen III synthase; catalyzing the sixth step in the tetrapyrrole biosynthetic pathway	Ayliffe et al., 2009
Gain	Rice FOX hunting	Rice	17, 18	Forward	*GLK1*	GARP TF; key regulator of chloroplast development	Nakamura et al., 2009
Gain	Rice FOX hunting	Rice	17, 18	Forward	*TIFY11b*	JAsmonate ZIM-domain (JAZ) protein; regulating grain size through enhanced carbohydrate accumulation in stems	Hakata et al., 2012
Gain	Mini-scale Rice FOX hunting	Rice	19	Forward	*CPK21*	Calcium-dependent protein kinase; positive factor in the ABA and salt-stress signaling pathways	Asano et al., 2011
Gain	Rice FOX hunting	Rice	17, 18	Reverse	*ARAF1, ARAF3*	Arabinofuranosidases; hydrolyzing arabinose side chains from arabinoxylan, a major hemicellulose in monocots	Sumiyoshi et al., 2013
Gain	Rice FOX hunting	Rice	17, 18	Forward	*GTP1*	Protein containing GTP-binding and adaptin-binding domains; abiotic stress tolerance	Kurotani et al., 2015a
Gain	Rice FOX hunting	Rice	17, 18	Forward	*CYP94C2b*	Cytochrome P450 (CYP94C subfamily) protein; deactivating the bioactive JA-isoleucine (JA-Ile) conjugate and salt stress tolerance	Kurotani et al., 2015b
Gain	Rice FOX hunting	*Arabidopsis*	20	Forward	*BSR1*	Receptor-like cytoplasmic kinase (RLCK) family protein; resistance to both bacterial and fungal pathogens	Dubouzet et al., 2011
Gain	Rice FOX hunting	*Arabidopsis*	20	Forward	*HsfA2e*	Heat stress TF; heat-shock response	Yokotani et al., 2008
Gain	Rice FOX hunting	*Arabidopsis*	20	Forward	*SMCP1*	Small protein with a Ca^2+^-dependent lipid binding (C2) domain; tolerance to abiotic and biotic stresses	Yokotani et al., 2009
Gain	Rice FOX hunting	*Arabidopsis*	20	Forward	*CEST*	Chloroplast protein; abiotic stress tolerance possibly through protection against photooxidative damage	Yokotani et al., 2011
Gain	Rice FOX hunting	*Arabidopsis*	20	Forward	*JAmyb*	R2R3-MYB TF; JA-mediated abiotic and biotic stress responses	Yokotani et al., 2013

### Gain-of-function Resources by Overexpression

#### Activation Tagging

The concept of activation tagging by T-DNA was first described by Walden et al. (1994). In the system, multiple transcriptional enhancer elements are randomly introduced into the plant genome. Gene(s) adjacent to an insertion site of the enhancer elements display enhanced expression and show dominant gain-of-function phenotypes. The first successful example of activation-tagged gene was *Arabidopsis ICK1* encoding a histidine kinase homolog involved in cytokinin signaling (Kakimoto, 1996).

Activation tagging is applicable to both forward and reverse genetic screens, as is the case with loss-of-function resources generated by *Tos17, Ds*, and T-DNA insertions. Though activation tagging was first applied to generate gain-of-function resources in *Arabidopsis* (Weigel et al., 2000), numerous activation-tagged rice resources have been generated by the insertion of T-DNA or transposable elements (Jeong et al., 2002, 2006; Chern et al., 2007; Hsing et al., 2007; Mori et al., 2007; Qu et al., 2008; Wan et al., 2009). We first introduce two typical examples for elucidating gene functions using reverse genetic approach. Jeong et al. (2006) generated 47,932 activation-tagged lines by T-DNA and obtained 27,621 flanking sequence tags (FSTs) by inverse PCR. Using this resource and FST information, Lee et al. (2010) isolated an activation tagging line of the *OsCOL4* gene (*OsCOL4-D, D* for *Dominant*), a member of the *CONSTANS-like* (*COL*) family. Given that Cauliflower mosaic virus *35S* enhancer elements were inserted approximately 2 kbp upstream of *OsCOL4*, the transcript level was significantly upregulated in the line. *Oscol4* null mutants flowered early under short and long days. In contrast, *OsCOL4-D* plants flowered late in either environment, indicating that OsCOL4 functions as a flowering repressor in rice (Lee et al., 2010). Lee et al. (2012) analyzed the function(s) of OsYSL16, which is encoded by a member of 18 *Yellow Stripe1-like* (*YSL*) genes and may be involved in iron transport in rice. Knockout mutation and antisense suppression of *OsYSL16* did not alter Fe distribution and utilization. Two lines (*OsYSL16-D1* and *-D2*) ectopically overexpressing *OsYSL16* in the activation tagging population (Jeong et al., 2006) were identified. *OsYSL16-D* plants were more tolerant to iron deficiency than the wild type (WT), and OsYSL16 played a role in the mobilization of iron from roots to shoots (Lee et al., 2012). This is a typical example that function(s) of a gene of interest cannot be characterized by a loss-of-function mutation without altered phenotype(s) because of the possible functional redundancy, but can be characterized by gain-of-function mutation resulting in the altered phenotypes.

Next, three examples of the identification and characterization of genes by forward genetics using activation-tagged resources are shown. Mori et al. (2007) generated approximately 13,000 activation-tagged lines of rice using a binary vector carrying tetramerized *35S* enhancer elements along with *35S* minimum promoter and the first intron from the *phaseolin* gene in T-DNA with aim of activating flanking genes more strongly than by the enhancer alone. Among the population, a dominant lesion-mimic line, *Spotted leaf 18* (*Spl18*), was obtained by visual screening. *Spl18* showed enhanced resistance to both blast fungus and bacterial blight and harbored a T-DNA insertion 481 bp upstream of *OsAT1* encoding an acyltransferase. Increased expression of *OsAT1* was correlated with lesion mimicry and disease resistance (Mori et al., 2007). Nakagawa et al. (2012) identified a dominant mutation with short grain and semi-dwarfism (*Sg1-D*) by visual screening of the above-mentioned activation tagging population in rice (Mori et al., 2007). A gene with unknown function *Os09g0459200* located 1.4-kbp downstream of T-DNA insertion was overexpressed and responsible for the *Sg1-D* mutation. This mutant also showed erect, short, wide, and dark-green leaves and also decreased cell division and response to BR. The results indicated that SG1 down-regulates BR signaling or response to BR and causes suppression of cell proliferation and finally organ elongation (Nakagawa et al., 2012). Lu et al. (2014) developed a population of more than 200,000 activation-tagged rice lines based on T-DNA bearing tetramerized *35S* enhancers and identified a tagged line displaying enhanced drought tolerance by a forward genetic approach. In the mutant, T-DNA was inserted in the second intron of the *glutamate receptor-like 1* gene (*OsGLR1*). Another *GLR* gene *OsGLR2* was located adjacent to *OsGLR1*. Overexpressing *OsGLR1* or *OsGLR2* significantly enhanced drought tolerance in both rice and *Arabidopsis*, indicating that *GLR* genes play important roles in drought tolerance and could be used to improve agronomic performance of monocot and dicot crops (Lu et al., 2014).

Though the FOX-hunting system and CRES-T require fl-cDNA collection and cDNA clones of TFs, respectively, activation tagging can be applied to non-model plant species whose genome and/or cDNA sequences are not yet available. It is not realistic, however, to apply activation tagging in cereals without high-throughput transformation systems (Hiei et al., 2014). Accordingly, Ayliffe et al. (2007) applied activation tagging to barley, a non-model plant species, in combination with the *Ac/Ds* transposable element of maize. Approximately 20 primary transgenic barley lines carrying a modified *Ds* element (*UbiDs*) consisting of the terminal 5′ and 3′ ends of *Ac* and two copies of the maize *Polyubiqiuitin* (*Ubi*) promoter placed oppositely for ectopic overexpression of genes near T-DNA were each crossed to two *Ac* lines bearing the homozygous *Ubi* promoter::*Ac transposase*. In the hybrid progenies, transposition of the *UbiDs* element was observed at frequencies sufficient for mutagenesis. In a screen of 60,000 F2 seedlings, a mutant was identified that displayed necrotic phenotype with reduced seedling vigor. An *UbiDs* element was inserted downstream of the *uroporphyrinogen III synthase* (*Uros*) gene, whose product catalyzes the sixth step in the chlorophyll and heme biosynthetic pathway. In the mutant, *Uros* transcript was significantly reduced by the accumulation of antisense *Uros* transcript initiated by the *UbiDs* element (Ayliffe et al., 2009).

Although activation tagging is very useful for the identification and characterization of genes, as described above, caution is warranted. Activation-tagged lines sometimes show complex phenotypes, when the enhancer element affects the expression of several genes (Ichikawa et al., 2003; Tsuchida-Mayama et al., 2010). Besides acting as a gene-activating agent, the activation-tagging cassette can behave as an agent for gene disruption and antisense expression. Thus, activation tagging gives rise to both gain- and loss-of-function phenotypes, depending on the location and direction of insertions.

#### FOX Hunting

Because of remarkable progress in DNA sequencing technology and bioinformatics (Mochida and Shinozaki, 2010), whole-genome information of several grasses has recently been available, including rice (International Rice Genome Sequencing Project, 2005), maize (Schnable et al., 2009), sorghum (Paterson et al., 2009), *Brachypodium* (International Brachypodium Initiative, 2010), barley (International Barley Genome Sequencing Consortium, 2012) and wheat (International Wheat Genome Sequencing Consortium (IWGSC), 2014). Additionally, large-scale collections of fl-cDNA clones have been generated for rice (Rice Full-Length cDNA Consortium, 2003), barley (Mochida et al., 2009; Sato et al., 2009; Matsumoto et al., 2011), maize (Soderlund et al., 2009), wheat (Kawaura et al., 2009; Mochida et al., 2009), and *Brachypodium* (Mochida et al., 2013). Fl-cDNAs provide sequence information for mature mRNAs and proteins for the corresponding genes (Kondou et al., 2011). Thus, collections of characterized fl-cDNA clones are useful for analyzing functions of genes and proteins (Abdeeva et al., 2012). By exploiting an fl-cDNA collection of *Arabidopsis*, a novel gain-of-function technology was developed for the identification of functions of the genes and designated as FOX-hunting system (Ichikawa et al., 2006).

In the FOX-hunting system, individual fl-cDNAs are mixed at nearly equal molar ratios and then cloned downstream of a strong promoter in an expression vector. The resulting fl-cDNA expression library is transformed into plants via *Agrobacterium*, and a large population of transgenic plants can be obtained, many of which overexpress a single fl-cDNA. Among them, we can screen and isolate FOX lines displaying altered phenotypes of interest. Fl-cDNA(s) introduced in the FOX lines can be easily identified with T-DNA specific primers. In this manner, fl-cDNA that caused the altered phenotype(s) can be directly linked to a function. The FOX hunting system is applicable to various plants, including non-model species, provided that fl-cDNA clones and highly efficient transformation system are available (Ichikawa et al., 2006; Kondou et al., 2011).

In rice, excellent transformation systems using *Agrobacterium* are available (Hiei et al., 1994; Toki et al., 2006). Nakamura et al. (2007) and Hakata et al. (2010) have developed the FOX-hunting system in rice for the functional analysis of rice genes. Rice fl-cDNA clones were originally prepared and sequenced by two groups, the Institute of Physical and Chemical Research (RIKEN) and the Foundation for Advancement of International Science (FAIS) in Japan (Rice Full-Length cDNA Consortium, 2003). Nakamura et al. (2007) used a mixture of 13,980 rice fl-cDNA clones from RIKEN to construct a cDNA overexpression library for rice. Each fl-cDNA was cloned downstream of the maize *Ubi* promoter in a binary vector for overexpressing cDNAs. Approximately 12,000 rice FOX-rice lines were generated by transforming rice with the rice FOX-*Agrobacterium* library (Nakamura et al., 2007). Hakata et al. (2010) used 13,823 fl-cDNA clones from FAIS to generate a cDNA overexpression library. Each fl-cDNA was cloned downstream of the rice *Actin-1* promoter in a Gateway-based expression vector. Approximately 2,600 rice FOX-rice lines were produced (Hakata et al., 2010). Introduced fl-cDNA(s) in each line in these two FOX populations were identified by amplification of a fragment bearing fl-cDNA by genomic PCR using the promoter- and terminator-specific primers, followed by direct sequencing of the PCR fragment. A total of 7,050 fl-cDNAs were contained in the rice FOX-rice lines, of which 332 were found in both FOX populations.

Functions of several genes have been characterized using these two populations of FOX-rice lines. Nakamura et al. (2009) found FOX-rice lines producing green calli even in the presence of auxin, which suppresses plastid differentiation and greening. The mutation was caused by ectopic overexpression of a cDNA encoding a GARP TF, *Golden2-like 1* (*OsGLK1*). A large number of genes involved in chloroplast functions were highly expressed in the *OsGLK1*-FOX green calli. Well-developed thylakoid membranes and grana structures were observed in the plastids of *OsGLK1*-FOX calli. These results showed that OsGLK1 is a key regulator of chloroplast development (Nakamura et al., 2009). Hakata et al. (2012) identified FOX-rice lines overexpressing an fl-cDNA for OsTIFY11b/OsJAZ10 as a negative regulator of jasmonate (JA) signaling. The *OsTIFY11b*-FOX lines showed enhanced growth, including increased grain size, weight, leaf length, and plant height. The increases in grain size and weight were suggested to be caused by enhanced accumulation and translocation of carbohydrates in leaf sheaths and culms in the FOX lines (Hakata et al., 2012). As shown in these two examples, phenotype(s) altered by overexpression of each fl-cDNA for TF are useful clues to the function(s) of the gene product. Since duplicated *GLK* genes have functional redundancy, no altered phenotypes were observed in a single loss-of-function mutation in the two *GLK* genes (Wang P. et al., 2013).

A T1 population of 2,229 FOX-rice lines (Nakamura et al., 2007; Hakata et al., 2010) was treated with 300 mM NaCl for 5 weeks, and two lines showed salinity tolerance (Kurotani et al., 2015b). One of the two lines overexpressed an fl-cDNA for a protein, GTP1 containing putative GTP- and adaptin-binding domains, whose function remains to be elucidated. The other line carried an fl-cDNA encoding CYP94C2b, a cytochrome P450 family protein. CYP94C2b was involved in deactivation of the bioactive conjugate of JA, jasmonoyl isoleucine (JA-Ile) which activates JA signaling. *CYP94C2b*-FOX rice plants showed reduced responses to JA and enhanced shoot viability under salt stress, suggesting that the repression of JA action is responsible for enhanced salt tolerance (Kurotani et al., 2015a).

Asano et al. (2011) reported FOX-rice lines overexpressing calcium-dependent protein kinases (CDPKs), Ca^2+^-binding sensory kinases that regulate downstream components of calcium signaling. Because CDPKs constitute a large multigene family of 29 members in rice, biological functions of these *CDPK* genes would show functional redundancy and might be difficult to characterize by loss-of-function phenotypes. By using three *Agrobacterium* pools divided among 25 *CDPK* genes and the *OsCCaMK1* gene for a calmodulin-dependent protein kinase, Asano et al. (2011) applied a mini-scale FOX hunting system (Fujita et al., 2007). They produced a total of 250 *CDPK* FOX-rice lines accounting for 24 of the 25 *CDPK* and *OsCCaMK1* genes. Among FOX lines screened for salt stress tolerance, *OsCPK21*-FOX plants showed tolerance to salt stress by increasing sensitivity to ABA, suggesting the role of OsCPK21 as a positive factor in the ABA and salt-stress signaling pathways (Asano et al., 2011). This study is regarded as a test case for the mini-scale FOX hunting for a specific gene family. Considering the task of identification of fl-cDNA in each FOX line by genomic PCR and DNA sequencing, it might be convenient to transform host plants by fl-cDNA expression constructs one by one, when a multigene family consists of approximately 100 or fewer members. However, transforming rice with 1,000 fl-cDNA overexpression constructs one by one would be laborious. Provided that we generate an *Agrobacterium* library carrying 1,000 T-DNA vectors and transform rice with this single library, approximately 2,300 and 4,600 transgenic lines would require to cover 90 and 99% of fl-cDNAs, respectively, according to the equation formulated by Clarke and Carbon (1976). Although genomic PCR with a pair of specified primers followed by Sanger sequencing to identify individual fl-cDNA(s) in each transgenic line would be time-consuming, the FOX-hunting system would be a reasonable alternative to the one-by-one approach.

Sumiyoshi et al. (2013) reported FOX-rice lines overexpressing *OsARAF1* or *OsARAF3* for arabinofuranosidase (ARAF). ARAF dissolves arabinose side chains that bind to a xylose backbone and constitute a hemicellulose, arabinoxylan. Arabinose contents in the cell wall of *OsARAF1-* and *OsARAF3-*FOX lines decreased to 75.4 and 81.6%, and amounts of glucose increased to 128.2 and 134.2%, respectively, of that in WT. These FOX lines could be advantageous for producing bioethanol (Sumiyoshi et al., 2013). This is an example that FOX lines show valuable phenotypes of potential industrial use.

Kondou et al. (2009) developed a heterologous FOX-hunting system. An fl-cDNA expression library was constructed by individually placing approximately 13,000 rice fl-cDNAs under the control of the *35S* promoter. By introduction of fl-cDNA overexpression constructs into *Arabidopsis* via *in planta* transformation, more than 23,000 independent rice FOX-*Arabidopsis* lines, theoretically encompassing 11,000 of the 13,000 fl-cDNAs, were generated. The advantage of this heterologous FOX-hunting system is in characterizing gene functions using another host plant with a short life cycle and compact size that is equipped with a quick and efficient transformation system. Given that *Arabidopsis* is one of the ideal host plants that fulfill all the features, it was exploited for the heterologous FOX-hunting system to characterize gene functions (Kondou et al., 2009, 2011; Higuchi-Takeuchi et al., 2013).

Using approximately 20,000 rice FOX-*Arabidopsis* lines, Dubouzet et al. (2011) screened mutant lines resistant against *Pseudomonas syringae* and obtained 74 resistant lines. One of the selected genes, *BROAD-SPECTRUM RESISTANCE 1* (*BSR1*) encoding a receptor-like cytoplasmic kinase (RLCK) family protein, conferred strong resistance to both bacterial and fungal pathogens when overexpressed in both *Arabidopsis* and rice. Thus, the heterologous system could identify potentially useful gene(s) that confer multiple or broad-spectrum disease resistance on both dicot and monocot plants. Yokotani et al. (2008) screened the same population of rice FOX-*Arabidopsis* lines and isolated a mutant tolerant to heat stress. The rice fl-cDNA inserted in this line encoded OsHsfA2e, a member of heat stress TFs (HSFs) playing a central role in heat-shock response in many species (Yokotani et al., 2008). Yokotani et al. (2009) also screened 208 candidates for salt-tolerant lines among the FOX population. One of the mutants carried a rice cDNA encoding OsSMCP1, a small protein with a single C2 domain for Ca^2+^-dependent membrane binding. The *OsSMCP1*-FOX *Arabidopsis* plants showed improved tolerance to various abiotic and biotic stresses other than salinity (Yokotani et al., 2009). The other salt-tolerant lines carried an fl-cDNA for CHLOROPLAST PROTEIN-ENHANCING STRESS TOLERANCE (CEST), a novel chloroplast protein found in photosynthetic organisms (Yokotani et al., 2011), and JAmyb as a JA-responsive TF (Yokotani et al., 2013). These attractive examples show that the heterologous FOX-*Arabidopsis* resource is advantageous for high-throughput screening to yield plants with agronomically useful traits, although a homologous expression system would often be needed to confirm the function(s) of the gene of interest.

The heterologous FOX-hunting system could be a powerful tool for researchers working on non-model cereals, such as maize, barley and wheat, without appropriate facilities and skills of efficient transformation for those cereals but with large-scale collections of fl-cDNAs (Kawaura et al., 2009; Mochida et al., 2009; Sato et al., 2009; Soderlund et al., 2009; Matsumoto et al., 2011). *Arabidopsis* and rice with convenient transformation protocols would be suitable as host plants. Transgenic *Arabidopsis* plants can be efficiently generated with *in planta* transformation (Kondou et al., 2009). Accordingly, we need not consider background mutations caused by somaclonal variation, indicating the excellent linkage between mutant phenotypes and introduced cDNAs in the FOX-*Arabidopsis* lines. In contrast, rice transformation needs tissue culture. There is a tendency that the frequency of somaclonal variation is related to the duration of tissue culture. Minimizing the duration could suppress somaclonal variation in rice (Toki et al., 2006), indicating that the employment of high-speed transformation is important.

Wu et al. (2015) generated wheat FOX-rice plants using 1,455 fl-cDNAs of TFs from bread wheat and its relatives. TF cDNAs were divided into three size fractions: <1 kbp, 1–2 kbp, >2 kbp. Each fl-cDNA fraction was transformed into a Gateway-based binary vector to construct three wheat TF-FOX *Agrobacterium* libraries. By transforming rice with the libraries, more than 15,000 transgenic rice plants (T0 generation) were generated. Among 10,496 T0 plants that set seeds, 1,562 lines (14.9%) showed altered phenotypes at the T0 stage. Wu et al. (2015) then used 5,232 T1 lines to screen for salt and osmotic stress tolerance and identified seven TFs that functioned in stress tolerance.

Some graminaceous plants have recently emerged as new attractive model species. *Brachypodium distachyon*, a wild grass species, is one such species. *Brachypodium*, as well as wheat and barley, belongs to Pooideae, one of the three subfamilies of agronomical importance in the grass family Poaceae (Kellogg, 2001). This plant is small (∼20 cm), self-fertile and long-day with a short life cycle of a few months, and grows in the same facilities as *Arabidopsis* (Draper et al., 2001; Opanowicz et al., 2008). The compact genome of *Brachypodium*, approximately 272 Mbp on five chromosomes, has been sequenced by the International Brachypodium Initiative (2010). Moreover, molecular and genetic tools have been developed, including efficient transformation system preferentially using immature embryos (Garvin et al., 2008; Alves et al., 2009; Thole and Vain, 2012). Approximately 24,000 T-DNA insertion lines have been generated as of October, 2015 at the USDA-ARS^2^, and approximately 5,000 lines at the John Innes Centre (Thole et al., 2012). Application of the homologous FOX-hunting system to *Brachypodium* and/or that of the heterologous system to wheat and barley would be convenient and advantageous for high-throughput and accurate elucidation of functions of genes in Pooideae.

*Setaria viridis* (green foxtail), a wild ancestor of *S. italica* (foxtail millet), has also become popular as an attractive model species for the study of C4 grasses. *S. viridis* combines such convenient traits as diploidy (*n* = 9), short life cycle (6–9 weeks), small size (∼20 cm) and self-fertility with prolific seed production (∼13,000 seeds per plant) (Brutnell et al., 2010; Li and Brutnell, 2011; Brutnell et al., 2015). *S. viridis* has a relatively small genome (∼510 Mbp), and the whole genome sequence is available (Bennetzen et al., 2012; Zhang et al., 2012). The C4 photosynthetic pathway in association with a specialized leaf structure (Kranz anatomy) confers high productivity on several major food crops and bioenergy grasses, including maize, sugarcane, sorghum, switchgrass, and *Miscanthus* that belong to the Panicoideae subfamily (Brutnell et al., 2010). Recently, efficient procedures for *Agrobacterium*-mediated transformation of *S. viridis* have been developed using embryogenic calli derived from mature seeds (Brutnell et al., 2010; Martins et al., 2015b; Van Eck and Swartwood, 2015). Floral-dip transformation of *S. viridis* has been successful with a transformation efficiency of 0.6% (Martins et al., 2015a), and this could accelerate functional genomics in C4 grasses. For example, a population of *S. viridis* plants transformed with an expression library for maize fl-cDNAs (maize FOX-*Setaria*) could facilitate identification of functions of genes for C4 photosynthesis.

### Loss-of-function Resources by Overexpression

#### Gene Silencing

Antisense RNA and RNAi technologies are based on RNA-induced gene silencing [in other words, posttranscriptional gene silencing (PTGS)]. In the process, double-stranded small RNAs are generated and mediate sequence-specific RNA degradation for a target gene. RNA-induced gene silencing has been adopted extensively to plants since 1980’s for elucidating functions of particular gene(s) (Horiguchi, 2004; Eamens et al., 2008). In spite of the technological versatility, populations of transgenic plants overexpressing a variety of antisense RNA or RNAi constructs have not been generated until recently, probably because of the availability of various loss-of-function resources as described in the previous section. Wang L. et al. (2013) produced three hairpin RNA (hpRNA) libraries, OsHP2, OsHP4, and OsHP6, using size-fractionated cDNAs (200–400 bp, 400–600 bp, and 600–1,000 bp, respectively) from various rice tissues. Each T-DNA construct carried an RNAi cassette placed under the control of *Ubi* promoter for overexpression. The hpRNA library OsHP4 was introduced into *Agrobacterium*, and transformed into rice to generate more than 6,000 transgenic hpRNA plants. Among them, approximately 3,000 lines were deduced to possess the entire T-DNA regions, and more than 30% of them displayed poor growth and/or sterility. About 48% of the T1 lines derived from fertile T0 plants showed diverse mutations, and the ratio was much greater than that (3.5%) of a population of T-DNA insertion lines. The results suggested effective silencing of the target genes by the hpRNA library, and a high potential of the rice RNAi population for genomewide gene identification (Wang L. et al., 2013).

microRNAs (miRNAs), small RNAs of 20–24 nucleotides in length, have been found in almost all eukaryotes and play important roles through post-transcriptional control of genes in regulating almost all biological and metabolic processes including cell differentiation, organ development, response to biotic and abiotic stresses (Sun, 2012; Zheng and Qu, 2015). The combined genome and transcriptome sequencing studies have identified 427, 713, and 321 miRNAs in *Arabidopsis*, rice and maize, respectively (release 21 of miRBase) (Kozomara and Griffiths-Jones, 2014). A miRNA family, *MIR528*, is distributed only in monocot species (Cuperus et al., 2011). Warthmann et al. (2008) chose the 254-bp precursor of rice *MIR528* (*OsMIR528*), modified its miRNA/miRNA^∗^ region to silence endogenous and exogenous target genes, and evaluated the silencing of three different rice genes. Selective silencing of the target genes in transgenic rice plants overexpressing each amiRNA construct was induced and stably inherited in the progenies. *MIR390* family is highly conserved in plants (Cuperus et al., 2011). For high-throughput production of the amiRNAs and their overexpression in monocots, a series of expression vectors carrying the *OsMIR390* precursor was constructed and validated in transgenic *Brachypodium* plants (Carbonell et al., 2015). Among the chimeric *OsMIR390*-based precursors tested, amiRNAs accumulated to high levels, was accurately processed when expressed from the *OsMIR390*-based precursor (*OsMIR390-AtL*) of which the distal stem–loop sequence (16 bp) was replaced with that (31 bp) of *Arabidopsis MIR390a* (*AtMIR390a*), and induced effective silencing of individual target genes. In combination with a web tool, Plant Small RNA Maker Site (P-SAMS), for automated design of amiRNAs (Fahlgren et al., 2016), binary vectors carrying the *OsMIR390-AtL* precursor enable direct cloning of various amiRNAs and could be used for generating large-scale amiRNA construct libraries for silencing genes in monocots (Carbonell et al., 2015).

#### Chimeric REpressor Gene Silencing Technology (CRES-T)

Transcription factor is a protein that binds to specific DNA sequences typically located in the 5′-upstream regions of protein-coding sequences of the target genes and plays key role(s) in the control of gene expression in growth, development, and response to environmental stimuli in plants (Riechmann et al., 2000; Zhang, 2003). Most of plant TFs constitute multigene families and functions of TFs that belong to a subfamily often show redundancy. Therefore, it would be difficult to elucidate TF functions by knock-out or knock-down of the TF gene(s) of interest (Mitsuda et al., 2011a). To overcome such functional redundancy in TFs, Hiratsu et al. (2003) developed a novel gene silencing system designated as CRES-T. In this system, a TF is converted to a strong repressor (chimeric repressor) by fusion of the EAR-motif repression domain (SRDX). Overexpression of the chimeric repressor of the TF in host plants suppresses expression of target genes of both the TF and those with functional redundancy, and the dominant-negative phenotype(s) give clues to the function(s) of TF members (Hiratsu et al., 2003; Kubo et al., 2005; Mitsuda et al., 2011a).

To date, several TF genes have been characterized using CRES-T in rice (Mitsuda et al., 2006; Ogo et al., 2008; Mito et al., 2011; Tanaka et al., 2012; Todaka et al., 2012; Yoshida et al., 2013). For instance, Ogo et al. (2008) identified a novel TF of the NAC family IDEF2 that specifically binds to the iron deficiency-responsive *cis* element 2 (IDE2) in rice and barley. Transgenic rice plants in which the function of IDEF2 was disrupted by RNA interference (RNAi) or CRES-T (*IDEF2::SRDX* under the control of rice *Actin-1* constitutive promoter) revealed aberrant iron homeostasis and repression of expression of genes induced under iron deficiency, indicating that IDEF2 functions as a key TF regulating iron-deficiency response (Ogo et al., 2008).

Yoshida et al. (2013) found two NAC TFs in rice, SECONDARY WALL NAC DOMAIN PROTEIN 1 and 2 (OsSWN1 and OsSWN2), that are orthologs of NAC SECONDARY WALL THICKNINGS FACTORs (NSTs) from *Arabidopsis* as master regulators of secondary wall formation. *OsSWN1* transcription was highly active in sclerenchyma cells of leaf blades and less active in xylem cells. In contrast, *OsSWN2* transcription was particularly active in xylem cells and less in sclerenchyma cells. *OsSWN2* produces two alternatively spliced variants *OsSWN2L* and *OsSWN2S* of which the truncated ORF lacks a potential transcriptional activation domain. Transgenic rice plants expressing a chimeric repressor of *OsSWN2S* (*OsSWN2S-SRDX*) driven by the *OsSWN2* promoter caused stunted growth and needle-like shape and browning in leaves. Transgenic lines carrying *OsSWN2S-SRDX* driven by the *OsWN1* promoter showed drooping leaves, reduced thickness of secondary walls in sclerenchyma cells, and reduced lignin and xylose contents, resulting in enhanced cell-wall digestibility. These results show that OsSWNs regulate secondary wall formation in rice and would be useful for improving cereals and grasses for forage and industrial use of products, such as sugars and biofuels (Yoshida et al., 2013).

As described above, CRES-T is a powerful tool for characterization of various TFs even with functional redundancy. In ornamental and model plants, including torenia, chrysanthemum, cyclamen and *Arabidopsis*, a database of floral phenotypes induced by CRES-T has been developed (FioreDB ^3^) (Mitsuda et al., 2011b). For comprehensive study and discovery of unknown TF functions, a large population of CRES-T (TF cDNA::*SRDX* driven by the *35S* promoter) transgenic lines for 1,600 independent TFs from *Arabidopsis* was generated. Evaluation of CRES-T lines revealed that repressors for six TFs conferred high tolerance to salt and osmotic stresses on *Arabidopsis* plants (Kazama et al., 2013). CRES-T resources for TFs of monocots may be developed in some species, including rice and *Brachypodium*, with available collections of fl-cDNA clones.

In the Sections “Loss-of-Function Resources by Direct Modification of Genes” and “Gain- and Loss-of-Function Resources by Overexpression,” various loss- and gain-of-function resources are described with special emphasis on the overexpression resources (see Gain- and Loss-of-Function Resources by Overexpression). For comparison, advantages and disadvantages for respective mutant resources are summarized in **Table 3**.

**Table 3:** Comarison of artificial mutant resources in plants induced by various mutagens.

Mutagen/transgene	Overexpression of transgene(s)	Mutant genotypes	Typical phenotypes	Advantage	Disadvantage
Chemicals or irradiation	No	Recessive	Loss of function	Applicable to all species including non-model plants without efficient transformation systems. In addition to forward genetic screen, reverse genetic screen is applicable by using TILLING.	Seeds of M2 generation needed to observe mutant phenotypes. Mutants hard to obtain for essential genes for growth and development. Mutant phenotypes hard to recognize for genes constituting multigene families with functional redundancy.
Insertion of DNA fragments (ex. T-DNA, transposable elements)	No	Recessive	Loss of function	Applicable to both forward and reverse genetic approaches. Linkage between the mutant phenotype and the antibiotic resistance, and information on flanking sequence(s) of the inserted T-DNA facilitate the isolation of responsible gene.	T1^∗^ plants needed to observe mutant phenotypes. Not suitable for essential genes and those constituting multigene families with functional redundancy.
RNAi and amiRNA constructs (RNA silencing)	Typical	Dominant	Loss of function	Since mutant genotypes are dominant, loss-of-function phenotypes are observed in the transgenic regenerants (T0^∗^). Silencing of multiple genes is possible, if conservative sequences are present in the genes. For multigene families with functional redundancy, the possibility of recognizing mutant phenotypes may be higher than the induced and recessive mutants, because of the dominant nature.	Not suitable for essential genes.
Chimeric repressors of individual TFs (CRES-T)	Typical	Dominant	Loss of function	Highly effective even for TF genes which constitute multigene families with functional redundancy.	In principle, application limited to TFs. Not applicable to essential TF genes.
Insertion of enhancers (Activation tagging)	Essential	Dominant	Gain of function	Applicable to essential genes and those costituting multigene families with functional redundancy, and to both forward and reverse genetic approaches. Also applicable to various plants, if their transformation systems work well. An fl-cDNA collection not required.	Activation-tagged lines sometimes show complex phenotypes by the activation of multiple genes. They may display both gain- and loss-of-function phenotypes depending on the location and direction of T-DNA insertions. Ectopic overexpression of endogenous genes may sometimes cause unexpected alteration in the plants.
Insertion of fl-cDNAs [(Homologous) FOX hunting]	Essential	Dominant	Gain of function	Applicable to essential genes and those constituting multigene families with functional redundancy. Direct linkage between introduced fl-cDNA and the altered phenotype convenient for estimating gene function.	An fl-cDNA collection essential. Application limited to (model) plants with efficient transformation systems. Ectopic overexpression of an cDNA may sometimes cause unexpected alteration in the FOX plants.
Insertion of fl-cDNAs (Heterologous FOX hunting)	Essential	Dominant	Gain of function	Applicable to essential genes and those constituting multigene families with functional redundancy. Direct linkage between introduced fl-cDNA and the altered phenotype convenient for estimating gene function. Model species such as *Arabidopsis* and rice can be used as hosts.	An fl-cDNA collection essential. Ectopic overexpression of an cDNA may sometimes cause unexpected phenotypes in the heterologous FOX plants.

*^∗^Researchers working on cereal transformation tend to assign the generation of regenerated transgenic plants as T0.*

## Conclusion

Use of the gene-overexpression resources generated by activation tagging, FOX hunting, and CRES-T has resulted in the discovery and characterization of many cereal genes. When these resources are used for the identification, screening, and/or evaluation of genes and their functions of interest, the choice of appropriate resource(s), by considering their (plausible) expression patterns and networks and phenotypes, would be important. As DNA sequencing technology continues to progress, the cost and time required for whole-genome sequencing in many plants may be reduced. Then, collections of sequence-based resources, such as fl-cDNAs, can be developed for many plants. In cooperation with the development and improvement of transformation technology (Hiei et al., 2014), novel combinations of both homologous and heterologous FOX hunting systems may be developed. Numerous combinations of loss- or gain-of-function phenotypes with agronomically important traits may lead to “super cereals.” Most recently, genome editing technologies, including transcription activator-like effector nucleases (TALENs) and clustered regularly interspaced short palindromic repeats (CRISPR)/CRISPR-associated (Cas) system, have been developed in cereals (Zhang et al., 2014; Osakabe and Osakabe, 2015). By adequately combining homology-dependent recombination (i.e., gene targeting), the genome-editing technologies provide on-demand insertion, deletion, or replacement at the target locus in a genome. Therefore, novel plant materials may be generated, for example, a pinpoint targeted base substitution conferring a single amino-acid change, deletion, or substitution of a functional domain to alter the function and/or activity of a target protein (Endo et al., 2016; Schiml and Puchta, 2016; Sun et al., 2016). Appropriate combinations of classical and novel technologies and resources will thus provide novel materials for crop improvement and broaden the potential of cereal breeding.

## Author Contributions

All authors listed, have made substantial, direct and intellectual contribution to the work, and approved it for publication.

## Conflict of Interest Statement

The authors declare that the research was conducted in the absence of any commercial or financial relationships that could be construed as a potential conflict of interest.

## Footnotes

1 http://rmd.ncpgr.cn

2 http://jgi.doe.gov/our-science/science-programs/plant-genomics/brachypodium/brachypodium-t-dna-collection/

3 http://www.cres-t.org/fiore/public_db/
